# The effect of female breast surface area on skin stiffness and tactile sensitivity at rest and following exercise in the heat

**DOI:** 10.1113/EP091990

**Published:** 2024-08-22

**Authors:** Hannah Blount, Alessandro Valenza, Jade Ward, Silvia Caggiari, Peter R. Worsley, Davide Filingeri

**Affiliations:** ^1^ ThermosenseLab, Skin Sensing Research Group, School of Health Sciences The University of Southampton Southampton UK; ^2^ Sport and Exercise Sciences Research Unit, SPPEFF Department University of Palermo Palermo Italy; ^3^ PressureLab, Skin Sensing Research Group, School of Health Sciences The University of Southampton Southampton UK

**Keywords:** breast, exercise, female, skin mechanics, tactile sensation

## Abstract

Female development includes significant morphological changes across the breast. Yet, whether differences in breast surface area (BrSA) modify breast skin stiffness and tactile sensitivity at rest and after exercise in the heat remain unclear. We investigated the relationship between BrSA and skin stiffness and tactile sensitivity in 20 young to middle‐aged women (27 ± 8 years of age) of varying breast sizes (BrSA range: 147–502 cm^2^) at rest and after a submaximal run in a warm climatic chamber (32C ± 0.6C; 53% ± 1.7% relative humidity). Skin stiffness above and below the nipple and tactile sensitivity from the nipple down were measured. Associations between BrSA and both skin stiffness and tactile sensitivity at rest were determined via correlation analyses. Effects of exercise and test site were assessed by a two‐way ANOVA. Skin stiffness was positively correlated with BrSA 3 cm above the areola edge (*r* = 0.61, *P *= 0.005) and at the superior areola border (*r* = 0.54, *P *= 0.016), but not below the nipple (*P *> 0.05). The area 3 cm below the areola was also significantly stiffer than all other test sites (*P *< 0.043). Tactile sensitivity did not vary with BrSA (*P *> 0.09), but it varied across the breast (i.e., the area 3 cm below the areola was more sensitive than the inferior areola edge; *P *= 0.018). Skin stiffness and tactile sensitivity across the breast decreased after exercise by ∼37% (*P *< 0.001) and ∼45% (*P *= 0.008), respectively. These findings expand our fundamental understanding of the mechanosensory properties of the female breast, and they could help to inform sportswear innovation to better meet the support needs of women of different breast sizes at rest and following exercise.

## Abstract (highlights)

- **What is the central question of this study?**
  How do differences in breast surface area impact skin stiffness and tactile sensitivity in healthy women before and after running in the heat?
- **What is the main finding and its importance?**
  We found a new relationship between breast morphology and skin stiffness, such that skin stiffness increased linearly with breast surface area in the upper breast. Furthermore, skin stiffness and tactile sensitivity decreased after exercise in the heat. These findings expand our fundamental understanding of breast mechanosensory properties and they could help to inform sportwear innovation to meet the support needs of women of different breast sizes better.

## 1 INTRODUCTION

The breast is a complex part of the female anatomy that is highly deformable and, when unsupported, moves independently during dynamic movements, such as when running (Haake & Scurr, 2010; Nolte et al., 2016; Rajagopal et al., 2008). These dynamic breast movements can cause discomfort and represent a barrier to exercise for some women (Lawson & Lorentzen, 1990). As a result, >85% of women deem sports bras an essential item of clothing to support the breast and reduce the amount of breast movement (Gehlsen & Albohm, 1980; Lorentzen & Lawson, 1987; Scurr et al., 2010) and discomfort (Brown et al., 2014) during exercise. Nonethless, intrinsic support systems exist in the breast, with the primary one being the skin (Gefen & Dilmoney, 2007; Hindle, 1991).

The mechanical properties of human skin, including skin stiffness, are non‐linear, viscoelastic and highly variable with age, hydration, disease and anatomical site (Soetens et al., 2018). Skin stiffness is defined as the resistance to an external force that deforms the tissue from its original shape. Less stiff (i.e., softer) skin deforms at a higher rate at the point of contact than stiffer skin (Li & Gerling, 2023). It is well known that ageing causes changes in skin mechanical properties, reducing skin stiffness and elasticity (Escoffier et al., 1989), which can result in breast ptosis (‘dropping’) in females (Groyecka et al., 2017; Rinker et al., 2010). However, it has also been hypothesized that damage to the breast structure owing to repetitive stretch or strain experienced during exercise, or from lack of external support, could also lead to changes in the mechanical properties of local skin tissues (Page & Steele, 1999). Cyclical strain of *ex vivo* skin models has been shown to drive reductions in mechanical stiffness (Remache et al., 2018). Furthermore, increased tissue temperature owing to exercise has also been shown to reduce stiffness in a range of other soft tissues (Sapin‐de Brosses et al., 2010; Wu et al., 2001; Xu et al., 2008; Zhou et al., 2010). Owing to the differences in breast mass and corresponding strain, there might be a relationship between breast size, associated skin remodelling and consequent changes in skin stiffness at rest and after exercise.

Breast size varies greatly among women and can diverge over time owing to changes in body mass, menstrual phases, pregnancy, breast‐feeding and menopause (Azar et al., 2001; Wade et al., 2010). Variation in breast size can lead to differences in breast skin surface area (BrSA) and corresponding breast skin stretch, in addition to differences in breast volume and mass, which can, in turn, place further strains on the breast skin tissue. Norris et al. (2020) measured breast skin strain rates during static and dynamic activities and found that most females had peak values in the upper, lateral breast region when tested with no bra support. In the presence of high strain, collagen fibres in the skin are increasingly under tension, becoming uncrimped, which increases the stiffness of the tissue (Benítez & Montáns, 2017). Although this evidence indicates a relationship between the (breast region‐dependent) extent of breast movement and the resulting skin stiffness, there have been limited investigations on the relationship between breast size and breast skin stiffness both at rest and after exercise, despite the observed patterns of greater movement in larger breasts (McGhee & Steele, 2020; White et al., 2015).

Besides its role as an intrinsic support system of the breast, the skin also acts as a sensory organ to convey tactile sensations upon contact with mechanical stimuli (e.g., light touch) (McGlone & Reilly, 2010). Tactile sensations are a fundamental cutaneous sensory attribute that is necessary for sensing the external physical world, including one's interaction with clothing, such as the bra (Havenith, 2002; Song, 2011). Mechanoreceptors innervating the breast skin convey sensory inputs associated with feelings of pressure, itchiness, clinginess and comfort in an area that is almost always covered by a garment (Song, 2011). Previous research investigating breast sensitivity to tactile stimuli has indicated that larger breasts tend to have lower sensitivity (as evidenced by higher tactile detection thresholds) (Cornelissen et al., 2018; DelVecchyo et al., 2004; Tairych et al., 1998) and lower spatial acuity (Kasielska‐Trojan et al., 2021) than smaller breasts. However, the anatomical or physiological mechanisms underlying these breast size‐dependent changes in tactile sensitivity are yet to be confirmed. Notably, changes in skin stiffness at the fingertip have recently been proposed to play a role in size‐dependent changes in tactile sensitivity (Li & Gerling, 2023; Vega‐Bermudez & Johnson, 2004); however, whether the same stiffness‐dependent mechanisms apply at the breast and whether they contribute to modulate tactile sensitivity with increasing breast size remain unknown. It is also necessary to consider the impact of exercise on tactile sensitivity, because this sensory input might contribute to sports bra comfort. Previous research investigating the effect of exercise on cutaneous sensitivity has proposed an analgesic effect of exercise (Janal et al., 1984; Koltyn, 2000; Paalasmaa et al., 1991; Post et al., 1994; Valenza et al., 2019). However, whether exercise might induce changes in tactile sensitivity at the breast, in association with changes in skin mechanical properties, remains unknown.

Increasing our fundamental understanding of the role of breast size on skin mechanics and tactile sensitivity at rest and after exercise has valuable applications to inform the design of user‐centred sports bras that help to maintain skin health and improve the comfort of wearers of varying breast sizes. Hence, the first aim of this study was to characterize the relationship between breast size and skin stiffness over various breast regions, both at rest and after exercise in the heat. We hypothesized that larger breasts would present greater skin stiffness than smaller breasts, because of experiencing greater skin strain from dynamic breast movements. The second aim of this study was to characterize the relationship between breast size and tactile sensitivity, at rest and after exercise. We hypothesized that increased breast size would result in decreased tactile sensitivity, secondary to size‐dependent changes in skin stiffness. The third aim of this study was to determine the effects of exercise in the heat on changes in breast skin stiffness and tactile sensitivity. We hypothesized that exercise would result in decreased skin stiffness and tactile sensitivity, secondary to increased tissue temperatures and analgesic effects.

## 2 MATERIALS AND METHODS

### 2.1 Ethical approval

This study was approved by the University of Southampton Ethics Committee (approval no. 79007). All participants provided written informed consent prior to testing. The study conformed to the ethical standards set by the *Declaration of Helsinki*.

### 2.2 Participants

The study involved a convenience sampling approach of women with varying BrSA. Owing to the non‐linear association between BrSA and bra size (i.e., the latter being the most intuitive way of determining the breast size for eligibility purposes), four to six women for each bra‐size category, namely, small (corresponding size guide measurements of the bra: 32C–32E/34A–34C), medium (34D–34E/36A–36C), large (36D–36E/38A–38C) and extra‐large (38D–38E/40A–40C) were recruited. This purposeful approach aimed to achieve a wide range of BrSA. A total sample size of 20 healthy young women was targeted, in line with similar studies in the field (Sanchez et al., 2017; Sutradhar & Miller, 2013).

A total of 20 females of varying BrSA participated in the study (age, 26.7±7.7 years; weight, 72.2±13.3 kg; height, 170.6 ±4.8 cm; Table 1). Inclusion criteria included physically active women (i.e., performing 30 min of regular exercise of moderate intensity ≥3 days each week), free from musculoskeletal or neurological disease, not under any pharmacological treatment, with standard breast tissue type (i.e., no implants, reductions or mastectomy) and who fit size small, medium, large or extra‐large sports bras. They were also instructed to refrain from: (1) performing strenuous exercise in the 48 h preceding testing; (2) consuming caffeine or alcohol in the 24 h preceding testing; (3) consuming food in the 3 h prior to testing; and (4) applying creams or gels to the chest region. Menstrual phase was not ‘controlled for’, based on preliminary evidence that thermal sensation in females might not be modified independently by menstruation (Matsuda‐Nakamura et al., 2015). However, self‐reports of menstrual phase were collated. The participants were spread across a typical 28 day menstrual cycle (mean day of cycle, 13.6 ± 8.5), and two participants presented with irregular periods at the time of the study.

**TABLE 1:** Participant demographics (*n* = 20).

	Age (years)		Height (m)		Weight (kg)		BrSA (c $m^{2}$)	
Bra size	Mean	Min–Max	Mean	Min–Max	Mean	Min–Max	Mean	Min–Max
Small (*n* = 5)	24.0	18–30	1.68	1.63–1.72	59.0	56.4–66.7	170.0	147.2–230.1
Medium (*n* = 5)	22.8	19–27	1.69	1.65–1.70	67.5	59.8–76.1	246.3	203.5–288.0
Large (*n* = 6)	30.2	20–42	1.72	1.68–1.77	72.3	61.3–83.4	316.0	173.7–402.2
Extra‐large (*n* = 4)	29.8	21–44	1.75	1.66–1.83	94.1	87.0–97.9	432.7	300.0–502.2

Abbreviations: BrSA, breast surface area; Max, maximum; Min, minimum.

### 2.3 Experimental design

**FIGURE 1:**
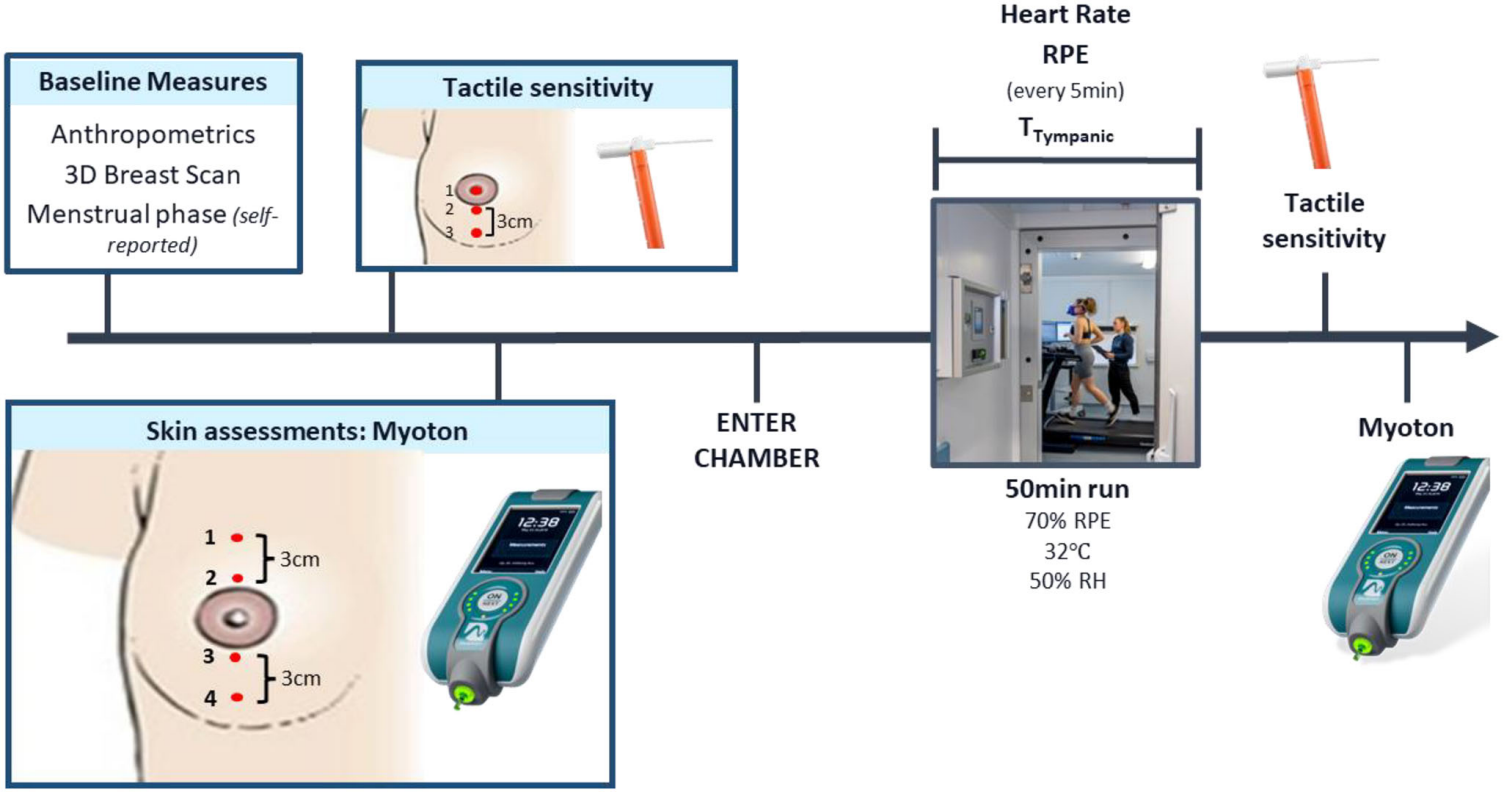
Schematic diagram of the experimental design. Abbreviations: 3D, three‐dimensional; 17RH, relative humidity; RPE, rating of perceived exertion; T_Tympanic_, body emperature measured at the tympanic membrane.

To establish breast size‐dependent differences in skin stiffness and tactile sensitivity at rest and following exercise, participants visited the laboratory on one occasion in a quasi‐experimental study design. First, BrSA geometry was captured, then skin stiffness and tactile sensitivity were assessed at rest, at multiple breast locations longitudinally down the breast and in line with the nipple (Figure 1). Assessments over multiple breast regions were deemed necessary to capture potential regional differences in skin properties and sensitivity (Norris et al., 2020; Valenza et al., 2023). For skin stiffness assessments, two areas above and below the nipple–areola complex were selected, to correspond to higher (above nipple) and lower (below nipple) strain areas (Norris et al., 2020). Tactile sensitivity assessments were also performed on the nipple–areola complex and at two sites in the lower breast. The nipple–areola complex has been shown to be the anchor of breast sensitivity (Kasielska‐Trojan et al., 2021), whereas the lower breast is an area more commonly associated with pressure discomfort when wearing a sport bra (Gho et al., 2010). This regional assessment also enabled skin stiffness and tactile sensitivity associations in the lower breast.

Following assessments at rest, participants wore standardized running shorts and a sports bra. They also used their own trainers and socks to perform a 50 min run in a climatic chamber set to 32C±0.6C and 53% ±1.7% relative humidity. Following the termination of the exercise, skin stiffness and tactile sensitivity at the breast were measured again according to the same procedures (Figure 1). We selected an exercise protocol performed in the heat because the stiffness and sensitivity data were collected as part of a larger project also investigating breast size‐dependent changes in sweat gland function and output, for which a combination of endogenous (i.e., exercise) and exogenous (environmental heat) thermal loads was required.

### 2.4 Experimental procedures

Participants were instructed to drink 500 mL of water 2 h prior to testing to ensure hydration during exercise. Upon arrival at the laboratory, participants provided a urine sample to measure urine specific gravity (Digital refractometer, KERN, Balingen, Germany). If urine specific gravity was >1.025 g/mL, participants were provided with 500 mL of water and tested again after 30 min before proceeding with the protocol, to avoid the risk of dehydration (Casa et al., 2005).

Initially, participants completed a questionnaire to report estimated menstrual phase and contraceptive use. Anthropometric measures of height, weight and BrSA were taken in a thermoneutral laboratory (21C±1.5C and 37% ± 5.2% relative humidity). Height was measured with a wall stadiometer and weight with a precision scale (KERN 150K2DL, Balingen, Germany). Participants were then asked to adopt a four‐point prone position such that the breasts could hang freely away from the torso, thus allowing a scan of the entire breast skin surface, using a precalibrated three‐dimensional (3D) white‐light surface scanner (EinScan H, Shining 3D Tech. Co. Ltd., Hangzhou, China; surface height accuracy of 0.05 mm). Markers were placed around the breast border based on a validated breast volume model (Göpper et al., 2020), from which surface area could be estimated using MeshLab (Visual Computing Lab, CNR‐ISTI, Pisa, Italy).

After the 3D scan, participants were asked to lie in a supine position, and tactile sensitivity assessments were performed. Although tactile sensitivity was the secondary outcome of this study, it was tested before the skin stiffness assessment to minimize perceptual sensitization attributable to mechanical stimulation from the skin stiffness assessments (Banik & Brennan, 2008). Tactile sensitivity assessments were performed on the nipple, at the base of the areola and 3 cm below, both in line with the nipple (Figure 1). Test sites were marked with a washable marker, and participants were familiarized with the procedure using the index finger as a practice body site. To evaluate sensitivity, we calculated tactile detection thresholds (i.e., the smallest amount of skin indentation that can be reported reliably by a participant as a tactile sensation) for each breast region, which were assessed using a set of 20 calibrated von Frey's monofilaments (North Coast Medical, Inc., Morgan Hill, LA, USA). Tactile detection thresholds were determined using the up–down method (Chaplan et al., 1994; Dixon, 1965), whereby stimulation began using the smallest monofilament (0.008 g), with progressive incrementation based on feedback by the participant. Inability to perceive indentation led to stimulation with the next largest monofilament until perception was reported. At this stage, an up‐and‐down sequence between the just perceivable and the non‐perceivable monofilaments was conducted for a minimum of three reversals. Successful completion of the reversals led to test termination and the bending force (in grams) of the just‐perceivable monofilament was deemed as the tactile detection threshold. To minimize testing errors, the same investigator and instruments were used for all measurements in a thermal neutral laboratory. The order of site was randomized following a simple random allocation to minimize order effects.

Upon completion of the tactile sensitivity testing, skin stiffness was evaluated non‐invasively using a myotonometer (Myoton Pro, Myoton SA, Estonia). The MyotonPRO uses a triaxial accelerometer (3200 Hz sampling frequency) and was used to apply a small mechanical displacement parallel to the skin surface with a J‐shaped probe. To ensure fixed contact between the probe and skin, thin (0.1 mm) double‐sided stickers (10‐mm‐diameter sticker attached to the disc) were used. For each probe, an initial force was exerted on the skin surface (0.18 N), and an additional mechanical force (0.4 N) for 15 ms, with a quick release, was applied on the skin surface to induce local deformation. The resultant damped natural oscillations caused by the viscoelastic properties of the tissue were captured with an inbuilt accelerometer. Skin stiffness and elasticity were estimated based on the oscillatory tissue response. This device has shown high reliability for muscle and skin stiffness assessment (John et al., 2023; Rosicka et al., 2021). Skin stiffness was assessed in four locations across the right breast (3 cm above the areola edge, the superior and inferior areola edge and 3 cm below the areola edge; all in line with the nipple), with mechanical impulses applied tangentially to the skin, five times, in a caudal direction. Test sites were marked with a washable marker, and participants were familiarized with the procedure. A verbal warning was given then skin stiffness was measured.

Upon completion of both tactile and skin stiffness assessments, participants entered the climate chamber set to 32C ±0.6C and 53%± 1.7% relative humidity. They were required to perform a 50 min run at a self‐selected speed eliciting a rate of perceived exertion (RPE) of 13, or ‘somewhat hard’, using the Borg scale (Borg, 1998). After cessation of the run, participants towelled off any sweat and resumed a supine position. The tactile sensitivity and skin stiffness tests were then repeated according to the procedures described above, whilst in the climate chamber.

### 2.5 Statistical analysis

Data normality and homoscedasticity were assessed using the Shapiro–Wilk and Levene tests, respectively. Skin stiffness data were identified to be normally distributed, hence parametric tests were used for analysis. Tactile sensitivity data were identified to be non‐normally distributed, hence non‐parametric descriptors and tests were used. Statistical analysis was performed using the SPSS statistical analysis software package (v.28.1, SPSS, Chicago, IL, USA). Data are reported as the means and SD, and significance was set at *P *< 0.05.

Descriptive statistics for all parameters of interest, including exercise intensity and duration, were collated. To establish breast size‐dependent differences in skin stiffness and tactile sensitivity at rest, the relationships between BrSA and skin stiffness and between BrSA and tactile sensitivity were assessed using Pearson's correlation and Spearman's rank correlation analyses, respectively. Correlation coefficients were calculated separately for each of the skin sites tested. In the event of a statistically significant correlation, regression analyses were performed (parametric data) to determine the extent of a parameter change (e.g., skin stiffness) for a unit change in BrSA.

Not all our participants were able to complete the full 50 min exercise trial duration, as some participants required an earlier termination of the run owing to volitional fatigue. Thus, for the exercise‐effect analysis we divided the study cohort into ‘finishers’ (*n* = 15) and ‘non‐finishers’ (*n* = 5). Specifically, all participants in the extra‐large bra category (*n* = 4) and one participant in the large bra category were unable to complete the full 50 min run trial.

To quantify exercise‐induced differences in skin stiffness across the breast at each site, a two‐way repeated‐measures ANOVA, with pre‐ and postexercise and skin site as the independent variables, was used for the ‘finishers’. In the event of statistically significant main effects or interactions, post‐hoc analyses were conducted with Bonferroni correction. To investigate the effect of exercise and site on tactile sensitivity, a Friedman test (followed by post‐hoc Wilcoxon signed‐ranks tests with a Bonferroni correction) was applied.

## 3 RESULTS

### 3.1 Descriptive statistics for rest and postexercise data

Descriptive statistics on the range of variation in skin stiffness and tactile sensitivity across breast sizes at rest and following exercise are reported in Table 2. Average exercise intensity (RPE) and running speed for the ‘finishers’ were 12.3 ± 1.0 (corresponding to between ‘light’ and ‘somewhat hard’) and 7.2 ± 0.9 km/h, respectively.

**TABLE 2:** Descriptive statistics of skin stiffness and tactile sensitivity parameters at all test sites, as a function of bra size group, pre‐ and postexercise.

		Small		Medium		Large		Extra‐large	
		Pre‐exercise (*n* = 5)	Postexercise (*n* = 5)	Pre‐exercise (*n* = 5)	Postexercise (*n* = 5)	Pre‐exercise (*n* = 6)	Postexercise (*n* = 5)	Pre‐exercise (*n* = 4)	Postexercise (*n* = 0)
**Skin stiffness (N/m)**									
3 cm above areola edge	Mean	244	211	292	216	311	238	504	–
	SD	85	47	69	48	123	58	168	–
	Min–Max	157–373	166–269	243–411	148–281	205–521	193–332	258–626	–
Superior areola edge	Mean	248	216	268	193	305	225	406	–
	SD	72	46	61	30	101	40	121	–
	Min–Max	163–327	165–282	214–361	154–235	200–471	168–270	230–508	–
Inferior areola edge	Mean	280	202	236	177	285	200	316	–
	SD	77	46	39	37	59	42	107	–
	Min–Max	206–408	166–272	187–283	119–213	187–342	140–255	223–444	–
3 cm below areola edge	Mean	326	219	327	239	409	323	487	–
	SD	81	63	28	38	101	82	165	–
	Min–Max	272–467	144–307	304–372	197–280	240–508	213–418	289–680	–
**Tactile sensitivity (g)**									
Nipple	Median	0.07	0.40	0.07	0.40	0.28	0.40	0.235	–
	IQR	0.33	0.09	0.03	0.00	0.24	0.20	0.34	–
	Min–Max	0.04–0.40	0.04–0.40	0.02–0.40	0.04–0.40	0.07–0.40	0.07–1.40	0.04–0.40	–
Inferior areola edge	Median	0.16	0.16	0.16	0.40	0.40	0.40	0.38	–
	IQR	0.33	0.14	0.12	0.24	0.70	1.00	0.68	–
	Min–Max	0.02–0.40	0.04–0.60	0.008–0.40	0.008–0.40	0.008–1.00	0.04–1.40	0.008–1.40	–
3 cm below areola edge	Median	0.008	0.045	0.07	0.07	0.24	0.07	0.28	–
	IQR	0.00	0.073	0.15	0.38	0.82	1.33	0.24	–
	Min–Max	0.008–0.40	0.02–0.16	0.008–0.16	0.008–0.40	0.008–1.00	0.008–1.40	0.16–0.40	–

Abbreviations: IQR, interquartile range; Max, maximum; Min, minimum.

### 3.2 Breast size‐dependent differences at rest

**FIGURE 2:**
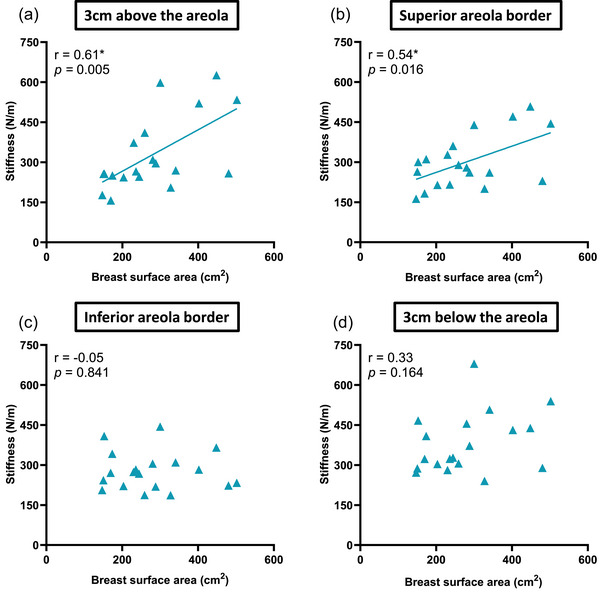
Relationship between breast surface area and skin stiffness at rest at: (a) 3 cm above the areola; (b) the superior areola border; (c) the inferior areola border; and (d) 3 cm below the areola. *Significant correlation (*P *< 0.05).

Regarding skin stiffness at rest (Figure 2), there was a statistically significant positive correlation with BrSA for sites 3 cm above the areola edge (*r* = 0.61, *P *= 0.005) and at the superior areola border (*r* = 0.54, *P *= 0.016), but not at the inferior areola border (*r* = −0.05, *P *= 0.84) nor at 3 cm below the areola edge (*r* = 0.33, *P *= 0.16). Regression analyses indicated that skin stiffness increased by ∼111 N/m at 3 cm above the areola edge [stiffness = 111.0 + 0.78(BrSA); *R* ^2^ = 0.38, *P *= 0.005] and by ∼163 N/m at the superior areola border [stiffness = 163.2 + 0.49(BrSA); *R* ^2 ^= 0.30, *P *= 0.016], for every unit change in BrSA.

**FIGURE 3:**
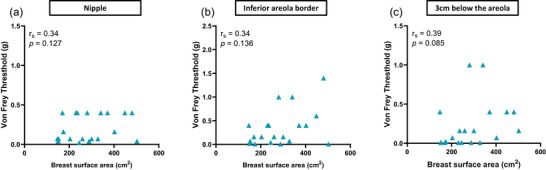
Relationship between breast surface area and tactile sensitivity thresholds at rest at: (a) the nipple; (b) the areola border; and (c) 3 cm below the areola.

The results for tactile sensitivity at rest revealed no correlation with BrSA at the nipple (*r* = 0.34, *P *= 0.13), at the areola edge (*r* = 0.34, *P *= 0.14) or at 3 cm below the areola (*r* = 0.39, *P *= 0.09) (Figure 3).

### 3.3 Effects of skin site and exercise

**FIGURE 4:**
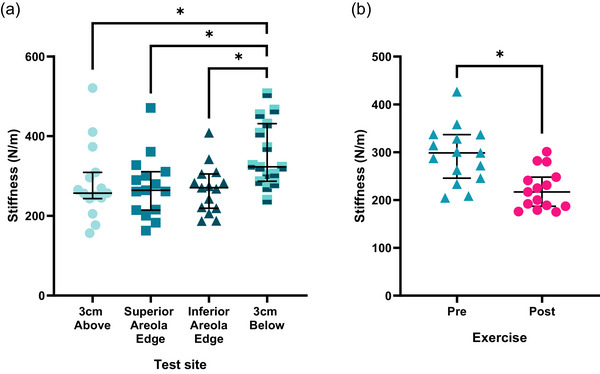
The main effect of (a) test site and (b) exercise on skin stiffness in the ‘finishers’ (*n* = 15). *Significant difference (*P *< 0.05).

Results of the two‐way ANOVA showed a significant effect of skin site [*F*(3,42) = 10.276, *P *< 0.001] and exercise [*F*(1,14) = 68.409, *P *< 0.001] but no significant interaction effect [*F*(3,42) = 1.066, *P *= 0.374] on skin stiffness in the ‘finishers’. When considering the overall effect of skin site, the findings revealed that the site corresponding to 3 cm below the areola had statistically greater skin stiffness than the sites corresponding to 3 cm above the areola {mean difference = +55.0 N/m [95% confidence interval (CI) = +1.2, +108.7]; *P *= 0.043}, the superior areola site [+64.6 N/m (95% CI +12.4, +116.7); *P *= 0.012] and the inferior areola site [+77.0 N/m (95% CI +30.3, +123.7); *P *= 0.001]. No statistically significant differences were found between the sites 3 cm above the areola and the superior and inferior areola (*P *= 1.00). Regarding the main effect of exercise, this decreased skin stiffness by 72.6N/m (95% CI = +53.8, +91.4; *P *< 0.001), corresponding to a percentage reduction in skin stiffness of ∼25% (Figure 4).

**FIGURE 5:**
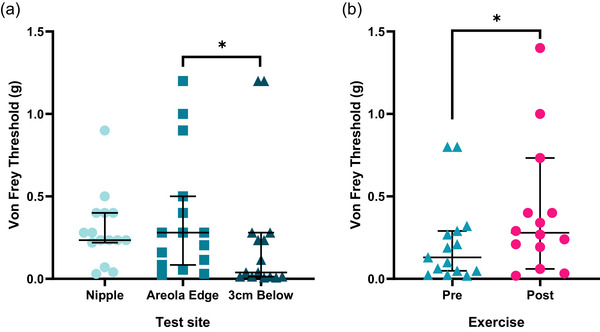
The effect of (a) test site and (b) exercise on tactile sensitivity thresholds in the ‘finishers’ (*n* = 15). Presented as median and 95% confidence interval. *Significant difference (*P *< 0.05).

The results of Friedman's test indicated a main significant effect of test site and exercise on tactile sensitivity [χ^2^(5) = 22.86, *P *< 0.001]. When considering the overall effect of skin site, the site corresponding to 3 cm below the areola had statistically lower tactile thresholds (−0.14 g; *P *= 0.018) and therefore greater tactile sensitivity than the inferior areola site. No statistically significant differences were found between the nipple and inferior areola edge (*P *= 0.55) and 3 cm below the areola (*P *= 0.27). Regarding exercise, this increased tactile thresholds by an average of 0.21 g (*P *= 0.008), corresponding to a percentage reduction in tactile sensitivity of ∼45% (Figure 5).

## 4 DISCUSSION

The first aim of this study was to characterize the relationship between BrSA and skin stiffness over various skin regions, at rest and following exercise. The second aim was to characterize the relationship between breast size and tactile sensitivity, at rest and following exercise. The third aim was to determine the effects of exercise in the heat on changes in breast skin stiffness and tactile sensitivity. Our findings partly supported our primary hypothesis that skin stiffness increases with greater breast size. However, this was observed only at the breast site above the nipple. Regarding our secondary hypothesis, our findings did not demonstrate a relationship between breast size and skin tactile sensitivity. Finally, our findings supported our third hypothesis, in that we recorded a postexercise decrease in skin stiffness and tactile sensitivity of ∼37% and 45%, respectively. These findings highlight a new relationship between breast morphology and local skin stiffness, in addition to the effects of exercise on skin properties and tactile sensitivity. These results provide new fundamental and applied insights on breast mechanical and sensory properties, which could inform future research and apparel design considerations, as discussed below.

### 4.1 Breast surface area and skin stiffness

We had hypothesized that females with larger BrSA would demonstrate greater skin stiffness owing to greater mechanical strain in these load‐bearing tissues (Norris et al., 2020). This was confirmed in the upper breast region, with about one‐third of the variance in skin stiffness being explained by BrSA. However, this was not consistent across breast sites; for example, we did not observe this relationship at the sites below the nipple line. The observed site differences in skin stiffness in relationship to BrSA are probably attributable to variability in breast skin strain (Norris et al., 2020). It has been shown previously that breast skin strain rates during static and dynamic activities are greatest in the upper, lateral breast region when tested with no bra support. Greater skin strain rates tend to occur in the vertical plane, owing to gravitational pulling (Choo et al., 2010). It has also been identified previously that breast skin thickness decreases with larger breast size (Willson et al., 1982), and thinner skin can have lower tensile strength than thicker skin (Hussain et al., 2013). The upper breast region might be more susceptible to this combined effect of thinner skin and greater strain rates as this region supports more of the breast mass. When collagen fibres in the skin are increasingly under tension and strain, fibres become uncrimped, which increases the stiffness of the tissue (Benítez & Montáns, 2017). These considerations might provide an explanation for the stiffness–size relationship observed in the upper breast. It is important to note that our findings are in contrast to the skin mechanical changes observed owing to prolonged skin stretch in obese populations (Choo et al., 2010; Ibuki et al., 2018; Smalls et al., 2006), where significant reductions in skin stiffness and elasticity have been revealed. A potential reason for these differences might be attributable to other factors, such as dermal thickness, subdermal tissue composition (i.e., subcutaneous body fat vs. breast tissue) and other comorbidities that might influence skin properties in obese people (Smalls et al., 2006).

### 4.2 Breast surface area and tactile sensitivity

In this study, no relationship was found between BrSA and tactile sensitivity in the lower breast region. In contrast, previous studies have demonstrated that smaller breasts tended to have higher tactile sensitivity (Cornelissen et al., 2018; DelVecchyo et al., 2004; Tairych et al., 1998) and greater spatial acuity (Kasielska‐Trojan et al., 2021) than larger breasts. However, these differences might be attributable to variations in the breast areas tested across studies. For example, Tairych et al. (1998) found that the largest size‐related differences (small vs. large breast) in tactile sensitivity occurred in the superior (small to large difference in Semmes–Weinstein value = 0.81), medial (0.86) and lateral (0.87) breast areas in comparison to, for example, the inferior (0.71) breast area, which was the primary area tested in the present study.

It is a limitation of the present study that we did not collect tactile sensitivity data over the upper breast. Tactile sensitivity has been shown to decrease with increasing stiffness at the fingertip (Li & Gerling, 2023; Vega‐Bermudez & Johnson, 2004). It is thought that if the skin fails to conform, or does so poorly, the mechanoreceptors responsible for detecting stimuli are not stimulated. Given our observation of increasing skin stiffness with BrSA at the upper breast, we could speculate that, had we assessed tactile sensitivity over this area, we might also have observed a similar reduction in tactile sensitivity to the one observed by Tairych et al. (1998). Clearly, further research is required to investigate the relationship between skin stiffness and tactile sensitivity across the whole breast.

### 4.3 Effects of exercise on skin stiffness and tactile sensitivity

In this study, we demonstrated that exercise under heat stress reduced breast skin stiffness and tactile sensitivity. This observation aligns with previous research into soft tissue biomechanics, which has identified that increases in tissue temperature, owing to exercise and the external environment, reduce stiffness in a range of soft tissues (Sapin‐de Brosses et al., 2010; Wu et al., 2001; Xu et al., 2008; Zhou et al., 2010). Increased temperature makes the skin more compliant, probably owing to collagen denaturation, which reduces the tensile strength of the tissues (Wall et al., 1999; Wright & Humphrey, 2002). However, it is important to note that the present study was not designed to delineate between temperature and exercise effects, owing to the combined nature of the intervention. Cyclic strain from rhythmic breast displacement during the run (Remache et al., 2018) or changes in skin hydration status (Berkey et al., 2021) attributable to sweating might also drive changes in skin stiffness after a bout of running in the heat. For example, increased skin hydration causes the stratum corneum to swell and reduces the elastic modulus of skin from ∼100–200 MPa to as low as ∼2 MPa (Berkey et al., 2021). Changes to the mechanical state of the upper stratum corneum layer subsequently change the mechanical environment of the deeper skin layers. This might lead to changes in the activation of afferent neurons innervating the skin and modify perceptions related to skin deformations (Bennett‐Kennett et al., 2023). It would, therefore, be reasonable to expect that the exercise‐induced reduction in stiffness, along with an increase in skin hydration attributable to sweating, would increase tactile sensitivity (André et al., 2010, 2011; Dione et al., 2023; Li & Gerling, 2023; Vega‐Bermudez & Johnson, 2004). However, this is in contrast to our findings of a reduced tactile sensitivity postexercise. We therefore speculate that the observed postexercise loss of sensitivity might be explained by the central analgesic effects of exercise on tactile sensitivity (i.e., attenuation of neural responses) (Janal et al., 1984; Koltyn, 2000; Paalasmaa et al., 1991; Post et al., 1994), which might have been more prominent than the peripheral effect of reduced skin stiffness. Furthermore, it cannot be excluded that prolonged exercise might also have reduced cognitive performance, impacting the capacity of participants to focus on a tactile discrimination task (Donnan et al., 2021; Gaoua et al., 2011). Future studies should therefore consider an experimental design that more directly addresses the central versus peripheral effects of exercise on local skin sensitivity.

### 4.4 Limitations and future directions

This study has some limitations. First, using the nipple and areola as a reference point, with fixed distances for measurement, meant that in smaller‐breasted women, 3 cm from the areola edge could have been near the top of the breast or breast base, whereas in larger‐breasted women, these test sites fell mid‐breast tissue. This method was selected because breast‐specific acuity has previously been shown to be systematically biased to the nipple (Kasielska‐Trojan et al., 2021). However, because of this approach, differences in the subdermal tissue (breast mass vs. more bony structures) might have influenced the results. Future studies could consider outlining the breast border and measuring proportional distances from the nipple to the breast border instead of fixed distances. A further future point to consider in relationship to our method of breast measurement would be also to investigate the effect of breast volume and density. Larger or more protruding breasts might be subject to higher strains and skin stiffness, which we were unable to characterize using our measurement of BrSA, hence this might open further interesting investigations.

Second, the larger‐breasted females tended to be older than the smaller‐breasted participants. Overall, ageing causes the epidermis to become stiffer (Diridollou et al., 2001; Hamasaki et al., 2018). It has been shown that females experience lesser age‐related changes in skin stiffness than men, but the largest changes in skin stiffness at the face and cleavage have been shown to occur between the age of 20 and 40 years in females (Diridollou et al., 2001; Krueger et al., 2011). However, if the differences in skin stiffness across BrSA were an age‐related effect, this relationship would probably have been present at all skin sites, not only the upper breast.

A final point to consider is that skin stiffness measurements in this study were taken in a supine posture, where the breasts were not under tension owing to gravity. If taken in an upright posture, whilst the skin was under strain, there is a distinct possibility that we would have seen greater differences in skin stiffness owing to the action of gravity. Future research should consider measuring breast skin stiffness in different postures to improve our understanding of skin mechanical properties in this tissue.

## 5 CONCLUSION

The results of this study demonstrate a dynamic interplay between breast size, skin stiffness, tactile sensitivity and exercise. Breast size‐dependent differences in skin stiffness exist in the upper breast at rest, such that the larger the breast, the greater the skin stiffness. Further research is required to investigate whether this effect impacts tactile sensitivity in this region. Exercise in the heat led to reduced skin stiffness and tactile sensitivity to a meaningful extent (i.e., between ∼37% and 45%). These findings expand our fundamental understanding of mechanical and sensory properties of the breast. Furthermore, this knowledge could help to inform sports bra design that better meets the support needs of the skin of the upper breast in women of different breast sizes, both at rest and after exercise in the heat.

## AUTHOR CONTRIBUTIONS

Hannah Blount, Davide Filingeri and Peter Worsley conceived the initial outline for the article. Acquisition was completed by Hannah Blount, Alessandro Valenza and Jade Ward. Hannah Blount, Silvia Caggiari, Davide Filingeri and Peter Worsley contributed to interpretation. Hannah Blount, Davide Filingeri and Peter Worsley contributed to drafting the manuscript, and all authors approved the final version of the manuscript and agree to be accountable for all aspects of the work in ensuring that questions related to the accuracy or integrity of any part of the work are appropriately investigated and resolved. All persons designated as authors qualify for authorship, and all those who qualify for authorship are listed.

## CONFLICT OF INTEREST

None declared.

## Data Availability

Data will be made available upon publication at the University of Southampton data repository (https://eprints.soton.ac.uk/).
